# The Relationship between Alcohol Consumption and other Risk Factors Assessed Using An Ongoing Population-based Surveillance System

**DOI:** 10.3934/publichealth.2016.4.985

**Published:** 2016-12-01

**Authors:** Anne W Taylor, Zumin Shi, Eleonora Dal Grande, Creina Stockley

**Affiliations:** 1 Population Research & Outcome Studies, Discipline of Medicine, The University of Adelaide, South Australia, Australia; 2 Population Research & Outcome Studies, The Univeristy of Adealide, South Australia, Australia; 3 Health & Regulatory Information, The Australian Wine Research Institute, Urrbrae, Adelaide, South Australia, Australia

**Keywords:** alcohol, risk factors, surveillance, Australia

## Abstract

The aim of this study was to determine the relationship of alcohol consumption (reported in four different ways) with other specific disease-related risk factors (that is, smoking, high body mass index, low physical activity and insufficient fruit and vegetables). Data were collected from 2003 to 2015 in South Australia using an on-going monthly chronic disease and risk factor telephone survey of randomly selected persons (18+ years). The proportion of alcohol drinkers and, for those who drank alcohol, the proportion drinking more than one day per week, the proportion drinking on six or more days per week, and the mean alcohol drinks per day were assessed. Logistic regression and linear regression modelling were used on age and sex adjusted data. In total, over 71,000 respondents aged 18 years and older were interviewed (48.8% male). Overall prevalence of alcohol consumption was 81.3%. Trends in the direction promoted by current policies and preventative authorities were apparent with appropriate changes for all four measures for overall and for those underweight, undertaking sufficient activity, consuming <2 serves of fruit per day, consuming <5 serves of vegetables per day and with 3+ total risk factors. This research has provided evidence on the trends in alcohol consumption in relation to a range of other specific modifiable disease-related risk factors. The trends analysis has shown different patterns for each risk factor, and highlights the interplay between the respective modifiable or preventive risk factors.

## 1. Introduction

The clustering and inter-relation of un-healthy, modifiable, lifestyle behaviours such as harmful alcohol consumption, inadequate diet, cigarette smoking and low levels of physical activity, has been reported in both developed [1]–[5] and developing countries [6],[7]. These four major risk factors, together with a high body mass index (BMI), are related to major chronic diseases such as cancer, heart disease and diabetes [3]. Adopting healthy behaviours is seen as a long term strategy to controlling and preventing the chronic disease ‘epidemic’. Studies have shown the more risk factors present, the poorer the health outcomes [2],[4],[5],[7],[8] and the addition of a risk factor, rather than being cumulative, can have a multiplier effect on health outcomes [1],[9].

While smoking, low levels of physical activity and poor diet have shown clear associations with chronic diseases and resultant negative outcomes, the role of alcohol consumption, as a risk factor for ill-health, is still debated. Positive health (particularly cardiovascular) and psychosocial benefits are often reported with low to moderate alcohol consumption [10]–[12]. Harmful consumption of alcohol is associated with considerable health, societal and economic costs, and is a major public health concern [13]–[15]. These negative health outcomes include a range of cancers, cardiovascular disease and gastrointestinal diseases [16],[17]. Accordingly, public health efforts to limit harmful alcohol consumption are paramount.

There is only limited data relating to the interaction of alcohol consumption with other modifiable risk factors, and yet such data are important in the formulation of appropriate public health recommendations. One of the difficulties in this area of research is the multitude of ways in which alcohol intake is quantified [18]. These include self-reported drinking status (daily, weekly, usual, or weekend/work day), consumption (self-reported number of drinks, how often alcohol consumed on how many days, frequency of consuming five or more drinks per day, how often drink to intoxication or industry-based sales data), beverage specific consumption (often converted to estimations of grams of alcohol consumed) and scaled scores (e.g. AUDIT-C) [19].

The ongoing surveillance of risk factors for the major chronic diseases is important especially in regard to assessing the success or otherwise of major preventive efforts [20],[21]. A surveillance system undertaking repeated cross-sectional surveys of the same population over a long period of time is an important tool in measuring change, evaluating interventions and predicting future diseases rates [20]–[22], and facilitating the formulation of action plans [21],[23].

The aim of this research is to analyse data collected by the South Australian Monitoring and Surveillance System (SAMSS) [24] to determine the relationships of alcohol consumption (analysed in four different ways) with other specific disease-related risk factors (that is, smoking, high BMI, low physical activity and inadequate diet) over time. The research aims to assess the contribution and value of an on-going surveillance system in providing evidence in the differences in alcohol consumption when assessed by other risk factors.

## 2. Methods

SAMSS is an on-going monthly chronic disease and risk factor survey of randomly selected persons and has been collecting data monthly since July 2002 [24]. All households in South Australia with a telephone number listed in the Electronic White Pages (EWP) are eligible for selection in the sample. Each month, residential telephone numbers are randomly selected from the EWP. A letter introducing SAMSS is sent to the household of each selected telephone number. Within each household the person who had their birthday last is selected for interview. There is no replacement for non-contactable persons. Although surrogate interviews are undertaken on behalf of children, the analysis in this paper is limited to adults aged 18 years and over.

Data are collected by a contracted agency using Computer Assisted Telephone Interviewing (CATI) and interviews are conducted in English. At least ten call backs are made to the telephone number to interview household members. Calls are made seven days a week between 9 am and 8 pm. Replacement interviews for persons who cannot be contacted or interviewed are not permitted. Of each interviewer's work, 10% is selected at random for validation by the supervisor.

The SAMSS questionnaire has been approved by the South Australian Health Ethics of Human Research Committee (436.02.2014). Questions relating to alcohol included the two questions recommended by the [Australian] National Health and Medical Research Council [25]. These are “how often do you drink alcohol” and “on a day when you drink alcohol how many drinks do you usually have”. Both of these questions have been assessed for validity and reliability in the Australian CATI setting [26]. If the respondent indicated they did not drink alcohol they were not asked how many drinks they would usually have. For those who indicated they drink alcohol, they were prompted with “a standard drink is equivalent to a schooner or midi of full strength beer, a glass or wine or a nip of spirits” and then asked how many drinks they would usually have. The two questions allowed alcohol consumption to be presented in four ways and as such the proportion of alcohol drinkers and, for those who drank alcohol, the proportion who drank more than one day per week, the proportion drinking on six or more days per week, and the mean alcohol drinks per day were assessed in the analyses.

The other risk factor questions were current smoking status (current, ex and non-smokers recoded into current and ex/non), physical activity (derived on the amount of walking and moderate and vigorous activity in a one week period and recoded into no activity, activity but not sufficient and sufficient activity) [27], BMI which was derived from self-reported weight and height, and recoded into three categories (underweight/normal (BMI < 25), overweight (25 ≤ BMI < 30) and obese (BMI ≥ 30)) [28], and daily consumption of vegetables (0 to 4, 5+ serves) and fruit (0–2, 3+ serves) [29]. In addition, these five risk factors were cumulated and recoded into none to two and three or more risk factors.

The data are weighted by age, gender, area (metropolitan/rural) and probability of selection in the household to the most recent Australian Bureau of Statistics (ABS) Census or estimated residential population data so that the estimates calculated are representative of the adult population. Probability of selection in the household is calculated on the number of eligible people in the household and the number of listings in the EWP. The weights reflect unequal sample inclusion probabilities and compensate for differential non-response.

The current analysis used data collected in the period January 2003 to December 2015 for respondents aged 18 years and over. The data were age and sex adjusted. The mean response rate of SAMSS for this period was 59.1%. Data were analysed using STATA version 14. All estimates and analyses were conducted using *svy* in STATA to incorporate the sampling design. Logistic regression models were used to determine the trends associated with the various prevalence estimates, while linear regression modelling was used for the mean of alcohol consumption daily [30]. The trends were also presented in fractional polynomial plots by using monthly time points as a continuous variable [31]. Logistic regression parameters have been expressed in terms of odds ratios (OR) for clear readability using year as a continuous variable. The ORs detail the direction of the increase or decrease in trends. The logistic regressions were used to indicate direction with odds ratio of high/low alcohol consumption for year <1 indicating a decrease and >1 indicating increase. To determine seasonal trends, the prevalence of alcohol consumption pattern over time was estimated through seasonal autoregressive integrated moving average (SARIMA) models in STATA. These models are not plotted although positive seasonal trends are highlighted in the text.

## 3. Results

In total, 71,449 respondents aged 18 years and over were interviewed from January 2003 until December 2015. Overall, 0.3% (n = 191) of respondents refused to answer the alcohol consumption questions and were excluded from the analysis. In total, 48.8% of eligible respondents were male. Table 1 highlights the overall demographic characteristics of the respondents.

**Table 1.** Demographic profiles of respondents.

	n	% (95 CI)
**Age**		
18–39	25621	35.9 (35.5–36.2)
40–59	26107	36.5 (36.2–36.9)
60+	19721	27.6 (27.3–27.9)
**Gender**		
Male	34847	48.8 (48.4–49.1)
Female	36602	51.2 (50.9–51.6)
Employment *		
Full time	29648	41.5 (41.1–41.9)
Part time	14119	19.8 (19.5–20.1)
Unemployed	1935	2.7 (2.6–2.8)
Home duties	4341	6.1 (5.9–6.3)
Student	3365	4.7 (4.6–4.9)
Retired	15293	21.4 (21.1–21.7)
Unable to work	2661	3.7 (3.6–3.9)
Other	77	0.1 (0.1–0.1)
Country of birth *		
Australia	56402	78.9 (78.6–79.2)
UK/Ireland	7163	10.0 (9.8–10.2)
Other	7794	10.9 (10.7–11.1)
Marital status *		
Married/Living with partner	47463	66.4 (66.1–66.8)
Separated/Divorced	4877	6.8 (6.6–7.0)
Widowed	4260	6.0 (5.8–6.1)
Never Married	14760	20.7 (20.4–21.0)
**Household annual income**		
Up to $20,000	7583	10.6 (10.4–10.8)
$20,001–$40,000	10696	15.0 (14.7–15.2)
$40,001–$60,000	9173	12.8 (12.6–13.1)
$60,001–$80,000	8974	12.6 (12.3–12.8)
$80,001 or more	20993	29.4 (29–29.7)
Not stated	14029	19.6 (19.3–19.9)
**Total**	**71449**	**100.0**
**Education**		
No schooling to secondary	35700	50.0 (49.7–50.4)
Trade, certificate, diploma	19367	27.2 (26.8–27.5)
Degree or higher	16271	22.8 (22.5–23.1)
**Total**	**71338**	**100.0**

* Don't know/refused not report

**Figure 1.**
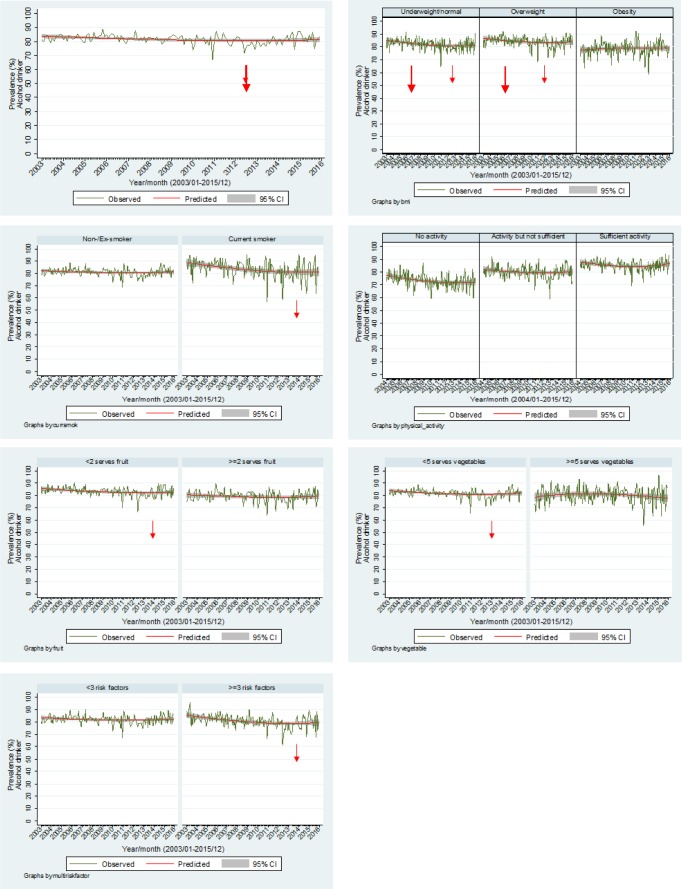
Overall alcohol consumption by other risk factors.

Figure 1 highlights the trends in alcohol consumption, overall, by the other five risk factors and by the variable assessing cumulative risk factors. The prevalence of alcohol consumption overall was 81.3% and decreased significantly over time (OR = 0.99, *p* = 0.003, 95% CI 0.98–0.99). The overweight BMI group had consistently higher prevalence rates of overall alcohol consumption while the trend decreased for the underweight/normal (OR = 0.98, *p* = 0.001, 95% CI 0.97–0.99) and overweight (OR = 0.99, *p* = 0.033, 95% CI 0.98–0.99) BMI groups over time. Current smokers had higher rates of alcohol consumption although the trend was significantly decreasing (OR = 0.96, *p* < 0.001, 95% CI 0.95–0.98). In terms of physical activity, those undertaking sufficient physical activity had consistent higher rates of alcohol consumption. There were no significant trends in alcohol consumption by activity level. Those consuming less than two fruits per day consistently showed higher alcohol consumption rates. The trend was decreasing significantly (OR = 0.99, *p* = 0.007, 95% CI 0.98–1.00). In terms of vegetable consumption, the trend for those consuming less than five vegetables per day was decreasing significantly (OR = 0.99, *p* = 0.007, 95% CI 0.98–1.00). For those respondents who had three or more disease-related risk factors, alcohol consumption decreased over time (OR = 0.98, *p* = 0.001, 95% CI 0.97–0.99). No seasonal effects were apparent.

**Figure 2.**
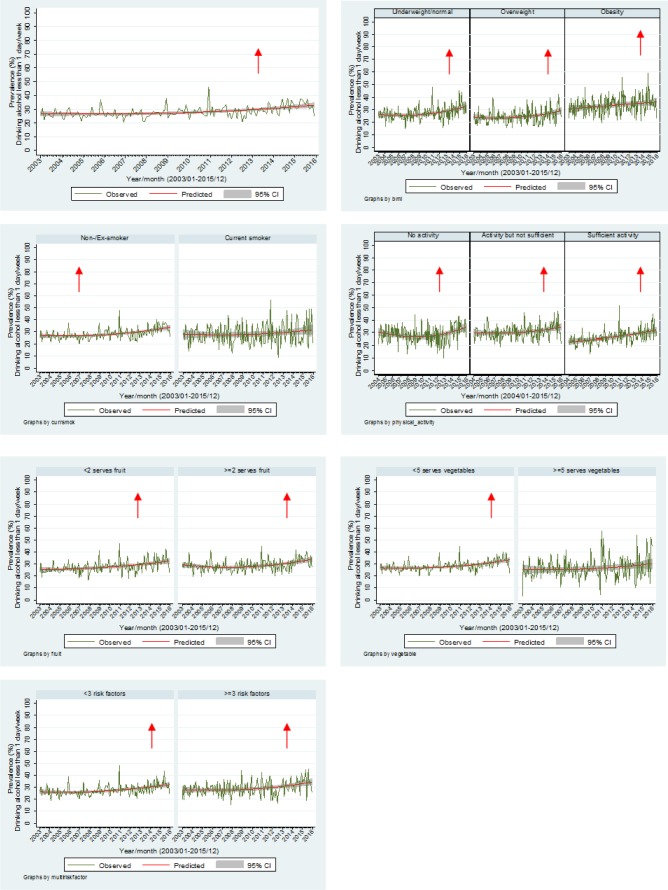
Proportion of adults drinking alcohol less than 1 day per week by other risk factors.

Figure 2 highlights the proportion of adults consuming alcohol less than one day per week with an overall prevalence estimation of approximately 30% with the trend increasing (OR = 1.03, *p* < 0.001, 95% CI 1.02–1.03). Increases in trends were also shown for the underweight/normal (OR = 1.03, *p* < 0.001, 95% CI 1.02–1.04), overweight (OR = 1.02, *p* < 0.001, 95% CI 1.01–1.03) and obese (OR = 1.02, *p* < 0.001, 95% CI 1.01–1.04) BMI groups; ex/non-smokers (OR = 1.03, *p* < 0.001, 95% CI 1.02–1.04); those undertaking no activity (OR = 1.02, *p* = 0.008, 95% CI 1.01–1.03), activity but not sufficient (OR = 1.02, *p* = 0.001, 95% CI 1.01–1.03) and sufficient physical activity (OR = 1.04, *p* < 0.001, 95% CI 1.03–1.05); those eating less than two fruits per day (OR = 1.03, *p* < 0.001, 95% CI 1.02–1.04) and those eating two or fruits per day (OR = 1.02, *p* < 0.001, 95% CI 1.01–1.03); those eating less than five vegetables per day (OR = 1.03, *p* < 0.001, 95% CI 1.02–1.04); and those in both the two or less risk factors (OR = 1.03, *p* < 0.001, 95% CI 1.02–1.03) and the three or more risk factor groups (OR = 1.03, *p* < 0.001, 95% CI 1.02–1.04). No seasonal effects were apparent.

**Figure 3.**
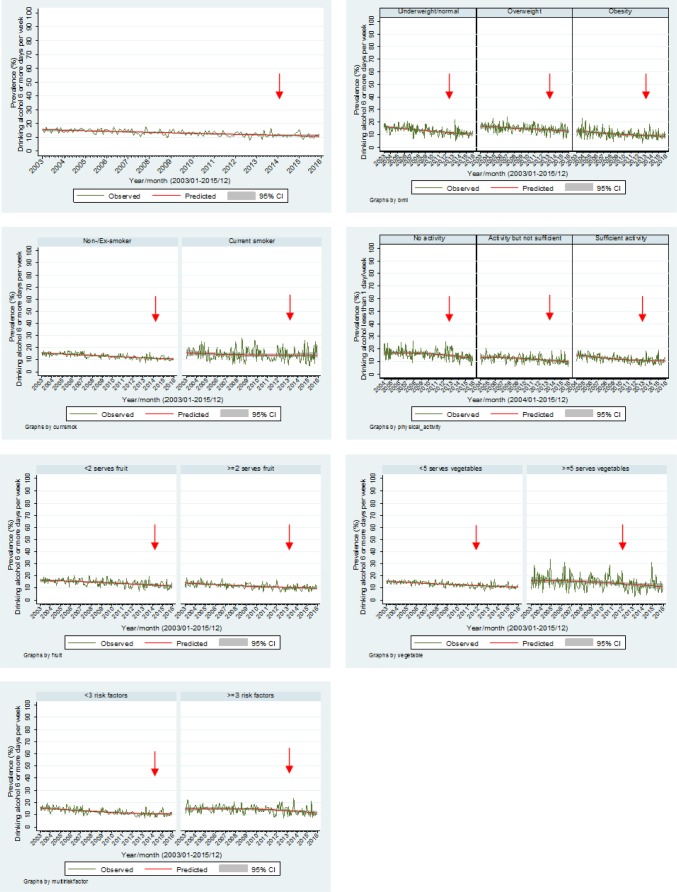
Proportion of adults drinking alcohol on 6 or more days per week by other risk factors.

Figure 3 highlights the trends for those consuming alcohol more than six days per week. There is an overall decrease in this trend over time (OR = 0.96, *p* < 0.001, 95% CI 0.95–0.97). Significant decreases in trends were found for all BMI groups (normal: OR = 0.95, *p* < 0.001, 95% CI 0.94–0.98), (overweight: OR = 0.97, *p* < 0.001, 95% CI 0.96–0.98), (obese: OR = 0.96, *p* < 0.001, 95% CI 0.94–0.98); non/ex-smokers (OR = 0.96, *p* < 0.001, 95% CI 0.95–0.97); smokers (OR = 0.98, *p* < 0.001, 95% CI 0.95–0.99); those undertaking no physical activity (OR = 0.98, *p* < 0.001, 95% CI 0.96–0.99), physical activity but not at a sufficient level (OR = 0.96, *p* < 0.001, 95% CI 0.95–0.97) and those undertaking sufficient physical activity (OR = 0.96, *p* < 0.001, 95% CI 0.93–0.99); those who consumed less than two fruits per day (OR = 0.96, *p* < 0.001, 95% CI 0.95–0.97); two or more fruits per day (OR = 0.96, *p* < 0.001, 95% CI 0.95–0.97), less than five vegetables per day (OR = 0.96, *p* < 0.001, 95% CI 0.95–0.97) and five or more vegetables per day (OR = 0.96, *p* < 0.001, 95% CI 0.94–0.98); and for both the two or less and the three and more cumulative risk factor groups (≤2 OR = 0.96, *p* < 0.001, 95% CI 0.95–0.97) (3+ OR = 0.97, *p* < 0.001, 95% CI 0.95–0.98). No seasonal effects were found.

**Figure 4.**
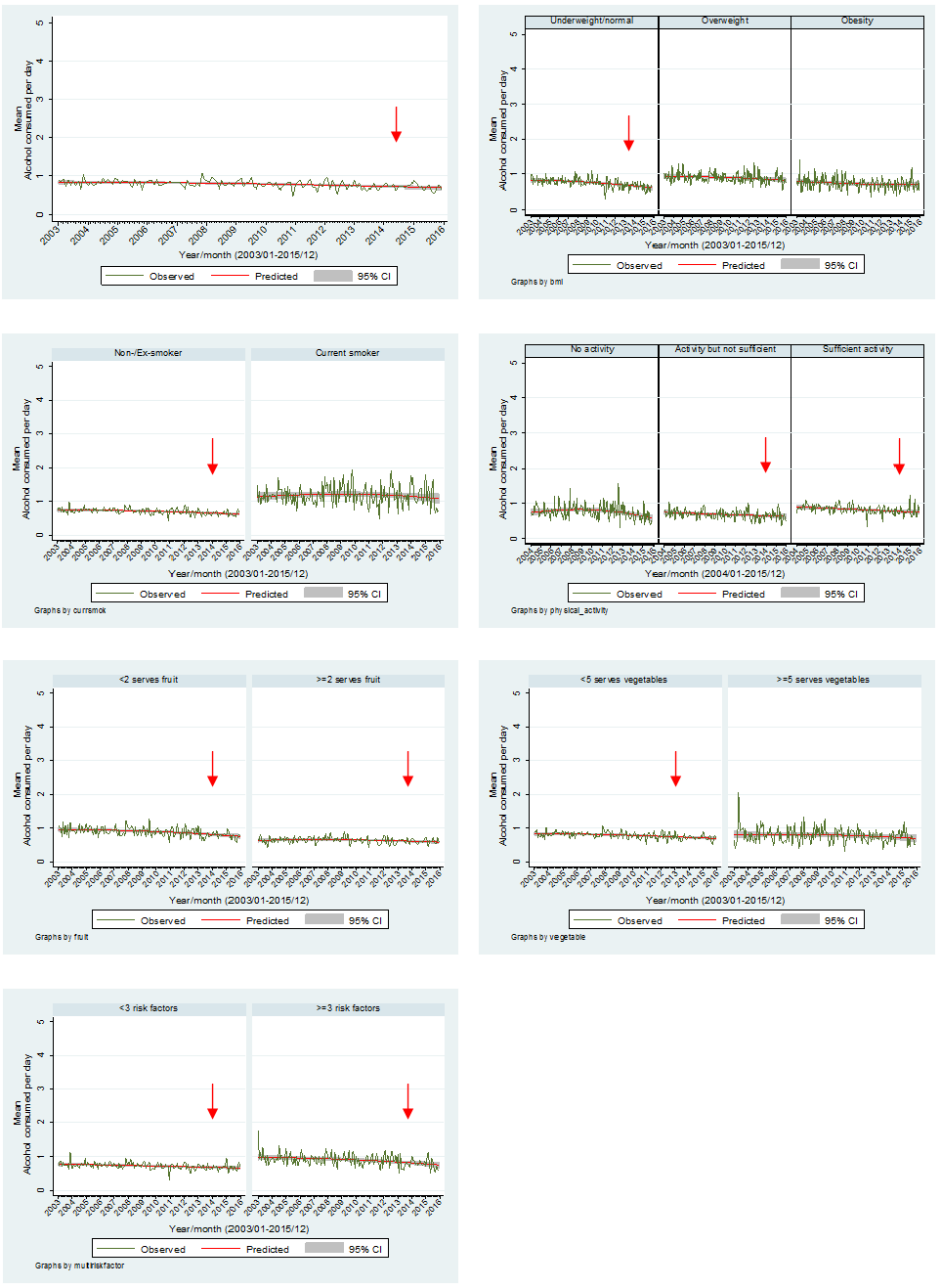
Mean number of alcoholic drinks per day by other risk factors.

Figure 4 highlights the mean number of drinks consumed per day for those who drink alcohol with consumption decreasing overall (*β* = −0.011, *p* < 0.001, 95% CI −0.014 to −0.007). The mean number of drinks per day significantly decreased for the underweight/normal group (*β* = −0.017, *p* < 0.001, 95% CI −0.021 to −0.012); non/ex-smokers (*β* = −0.009, *p* < 0.001, 95% CI −0.012 to −0.006); those undertaking physical activity but not at a sufficient level (*β* = −0.010, *p* = 0.001, 95% CI −0.016 to −0.004) and those undertaking sufficient physical activity (*β* = −0.013, *p* < 0.001, 95% CI −0.018 to −0.008); those who consumed less than two fruits per day (*β* = −0.015, *p* < 0.001, 95% CI −0.020 to −0.010), two or more fruits per day (*β* = −0.005, *p* = 0.013, 95% CI −0.009 to −0.001); less than five vegetables per day (*β* = −0.011, *p* < 0.001, 95% CI −0.015 to −0.008); and for both the two or less and the three and more cumulative risk factor groups (≤2 *β* = −0.008, *p* < 0.001, 95% CI −0.012 to −0.004) (3+ *β* = −0.014, *p* < 0.001, 95% CI −0.021 to −0.008). There were no seasonal effects.

## 4. Discussion

This research has provided evidence, using an Australian risk factor surveillance system, on the trends in alcohol consumption in relation to a range of other specific modifiable disease-related risk factors. The trends analysis has shown some differences in patterns for each risk factor and provides evidence that policy, programs and interventions that target harmful alcohol consumption and other risk factors are succeeding in decreasing alcohol consumption although some important differences are apparent. As argued by Berrigan et al [9], the interplay between the respective modifiable or preventive risk factors is an important requirement in assisting chronic disease prevention.

The overall prevalence of over 80% of this adult population consuming alcohol accords with published Australian national figures [32]. The downward trend in alcohol consumption over time is consistent with recent Australian national figures reporting a decrease in alcohol sales [33], overall consumption in the previous 12 months in the National Health Survey [34], the proportion of those 18 years or older consuming more than the recommended two standard drinks per day [34] and for 14 years of age and older an overall decrease in consumption [32].

Smoking is clearly related to a range of negative health outcomes including cancer and cardiovascular disease [35]. A relationship between smoking and moderate to heavy alcohol intake is well established [1],[9],[36]. While we find this relationship, we also see a trend for overall consumption of alcohol for current smokers to be decreasing and levelling to that of non/ex-smokers. Whether this is the consequence of successful policy endeavours is unknown although considerable public health effort has been expended in targeting the combined behaviours of smoking and excess alcohol consumption. Of concern is the lack of significant trends when the variables assessing alcohol consumption <1 day per week was assessed, which highlights appropriate increases/decreases for most other risk factors except for current smokers. This clearly continues to be a target population in terms of alcohol and smoking policies and interventions.

Low levels of physical activity are related to the risk of cancer, cardiovascular disease and diabetes [37]. The current study shows an association between sufficient physical activity and increased alcohol consumption. Despite some evidence to the contrary [36],[38],[39], other studies [40]–[42] including in Australia [43] have shown, as we did, that those undertaking sufficient physical activity were consuming the most alcohol and it remains to be determined what the relationship is between different types of physical activity [42],[44] and alcohol consumption. The socialisation and peer expectations associated with competitive and team sports can influence alcohol consumption [44]. Although those with higher levels of physical activity were consuming more alcohol, the trend is for decreased consumption in those obtaining a sufficient amount of physical activity.

There is no overall consensus in the data relating to the relationship between alcohol consumption and dietary habits [36],[43], although studies have shown a decrease in diet quality as alcohol consumption increases [45]. To some extent this appears to depend on the type of alcohol consumed with wine drinkers reported to have healthier diets than beer or spirit drinkers [36]. Types of alcohol consumed were not collected in this study. Analysis by socio-economic status to determine interactions is warranted. The ‘good’ news from this analysis is the consistent trends in the right direction for both levels of fruit and vegetable consumption.

The use of cross-sectional surveys at one point of time as a means of determining alcohol consumption has been criticised because of the wide seasonal variation in sales figures [46]. Continuous data collection systems where information is collected at least monthly, such as in the current study, have the capacity to demonstrate seasonal effects in alcohol consumption at the population level. While no seasonal effects were found in our analysis, seasonal effects in alcohol consumption have previously been shown for football players (post season) [47], those with allergic reactions [48] and associated with celebratory events (e.g. December) [49],[50]. The importance of knowing about these seasonal effects rests on the resultant increase in accident mortality, increased sick leave and societal effects such as increased alcohol-induced violence [49].

Weaknesses of this study include the self-reported nature of the data collection (with socially desirable responses possible because of the somewhat sensitive nature of the topic), lack of data on the type of alcoholic drink consumed (which has been shown to influence patterns of consumption [51],[52]) and lack of data on binge drinking. Although the cross-sectional nature of the data collection is also a weakness, the ability to produce time trends is a significant strength. As previously highlighted [53], the value of data collection occurring over many months lessens the likelihood of seasonal and weekly cycles. Nevertheless, studies have consistently shown that self-report data collected by surveys, whatever the mode, underestimate consumption when compared against point-of-sale data [53]. Notwithstanding, surveillance is more concerned about trends and consistency of associations rather than measurement prevalence *per se* and is beneficial in providing information on service requirements, policy directions and relationships with chronic diseases and other risk factors. A further weakness is the increased use of mobile telephones and decreased use of land-lines that could result in an under-representation of respondents (with younger and middle-aged persons more likely to have mobile telephones only and hence be excluded from sampling frames based on listed telephone numbers). The response rate of <60% could also produce biased data. Furthermore interviews were only conducted in English, which would limit the involvement of migrants from non-English speaking countries. Although some of the results of this study could be seen as ‘good news’, caution should be exercised in drawing too many conclusions. The lack of extensive details on possible confounders is also a limitation. No analysis was undertaken assessing the differences by demographic characteristics. Demographic differences in alcohol consumption was undertaken in a previous publication [54]. Other weaknesses have been documented more fully in a previous publication [54].

The strengths of this study include the use of a representative sample, with many previous studies having assessed the interrelation of risk factors using convenient samples. The large sample size, the range of alcohol-related variables able to be considered and the fact that the risk factors have been assessed using current recommended levels so that the study is providing evidence based on current public health recommendations, are additional strengths.

It appears that with many of the results presented in this manuscript that there are important changes in population-wide consumption of alcohol patterns. Trends in the direction promoted by current policies were numerous. Various studies that have assessed the clustering of risk factors have concluded that population-wide, multi-dimensional and integrated models and approaches should be implemented addressing multiple behaviours [4],[6],[8],[9] rather than a single factor approach. Although not often cited as the most problematic risk factor, harmful alcohol consumption can have major detrimental effects and as such research into the relationship of this risk factor with the other major risk factors for chronic diseases is of benefit to health promoters, public health and policy makers in determining appropriate preventive and intervention programs and strategies.
